# Distinguish fatty acids impact survival, differentiation and cellular function of periodontal ligament fibroblasts

**DOI:** 10.1038/s41598-020-72736-7

**Published:** 2020-09-24

**Authors:** Judit Symmank, Martin Chorus, Sophie Appel, Jana Marciniak, Isabel Knaup, Asisa Bastian, Christoph-Ludwig Hennig, Annika Döding, Ulrike Schulze-Späte, Collin Jacobs, Michael Wolf

**Affiliations:** 1 Department of Orthodontics, University Hospital Jena, Leutragraben 3, 07743 Jena, Germany; 2 Department of Orthodontics, University Hospital RWTH Aachen, Pauwelsstraße 30, 52074 Aachen, Germany; 3 Section of Geriodontics, Department of Conservative Dentistry and Periodontics, University Hospital Jena, Leutragraben 3, 07743 Jena, Germany

**Keywords:** Cell death, Cell growth, Orthodontics

## Abstract

Alveolar bone (AB) remodeling is necessary for the adaption to mechanical stimuli occurring during mastication and orthodontic tooth movement (OTM). Thereby, bone degradation and assembly are strongly regulated processes that can be altered in obese patients. Further, increased fatty acids (FA) serum levels affect bone remodeling cells and we, therefore, investigated whether they also influence the function of periodontal ligament fibroblast (PdLF). PdLF are a major cell type regulating the differentiation and function of osteoblasts and osteoclasts localized in the AB. We stimulated human PdLF (HPdLF) in vitro with palmitic (PA) or oleic acid (OA) and analyzed their metabolic activity, growth, survival and expression of osteogenic markers and calcium deposits. Our results emphasize that PA increased cell death of HPdLF, whereas OA induced their osteoblastic differentiation. Moreover, quantitative expression analysis of *OPG* and *RANKL* revealed altered levels in mechanically stimulated PA-treated HPdLF. Furthermore, osteoclasts stimulated with culture medium of mechanical stressed FA-treated HPdLF revealed significant changes in cell differentiation upon FA-treatment. For the first time, our results highlight a potential role of specific FA in the function of HPdLF-modulated AB remodeling and help to elucidate the complex interplay of bone metabolism, mechanical stimulation and obesity-induced alterations.

## Introduction

A balanced bone remodeling is critically important for the lifelong function of bone tissue as well as for the integration of intrinsic and extrinsic signaling information^1^. For example, mechanical forces during mastication or orthodontic treatment induce an adaption of alveolar bone remodeling processes according to force intensity and frequency^2–4^. This constant remodeling is characterized by the degradation and assembly of bone material based on a tightly controlled osteoblast and osteoclast cell differentiation and activity^1^. Imbalances are associated with diseases like osteoporosis and osteopetrosis^5^. Further, damages occurring during orthodontic procedures with excessive force can result in tooth root resorption or tooth loss due to excessive alveolar bone reduction^6,7^.

In obese patients, bone metabolism is altered but the complex relationship is still under investigation^8^. Clarifying the effects of being overweight is of great importance because obesity is a systemic heterogeneous disease with an increasing prevalence of affected people worldwide^9^. Patient and animal studies regarding the effects of obesity on bone metabolism partially resulted in somehow conflicting findings challenging the view on how overweight affect bone remodeling. However, high fat mass as well as hyperlipidemia were shown to negatively affecting bone mineral density and resulting in an increased risk for specific fractures^10,11^. Several animal studies and clinical investigations indicated that overweight is also correlated with enhanced alveolar bone loss^12–14^. An enhanced serum level in circulating fatty acids (FA) is one major characteristic of obesity, whereby the response of cells depends on the excess and type of FA^15,16^. While increased exposure to palmitic acid (PA) has been shown as being a negative regulator in various cellular contexts^17–20^, oleic acid (OA) appears to have a more beneficial cellular impact^17,21,22^. However, both fatty acids have been found to be increased in obesity and hypertriglyceridemia^23–28^. Thus, recent animal and in vitro studies revealed a greater bone loss in mice due to increased osteoclast numbers and activity as well as reduced osteoblast mineralization as a result of excessive exposure to the saturated PA compared to the unsaturated OA^29,30^. These findings led us to investigate, whether PA and OA overexposure impacts periodontal ligament fibroblasts (PdLF).

The periodontal ligament is the connective tissue between the cementum of the teeth and the alveolar bone and plays an important role in the healthiness of the periodontium^2,4^. During bone remodeling under physiological conditions, periodontal ligament fibroblasts can migrate towards the alveolar bone surface and differentiate to osteoblasts^31,32^. Moreover, PdLF are one major cell type modulating the differentiation and activity of osteoblasts and osteoclasts localized in the alveolar bone in response to mechanical strain application occurring during mastication and orthodontic treatment^2–4^. Thereby, PdLF contribute to bone remodeling through the regulation of relevant signaling mediators like RANKL and osteoprotegerin (OPG)^33^. A key molecular mechanism in osteoclastogenesis, the binding of RANKL to its receptor RANK, stimulates osteoclast maturation followed by bone resorption. OPG counteracts bone resorption by binding of RANKL and thereby preventing the activation of the RANK receptors^34^. Since these processes are relevant for a healthy bone remodeling, a possible impact of overweight and hyperlipidemia on PdLF function is of great interest in dental research.

## Results

### Palmitic acid, but not oleic acid, limits the survival of periodontal ligament fibroblasts

**Figure 1:**
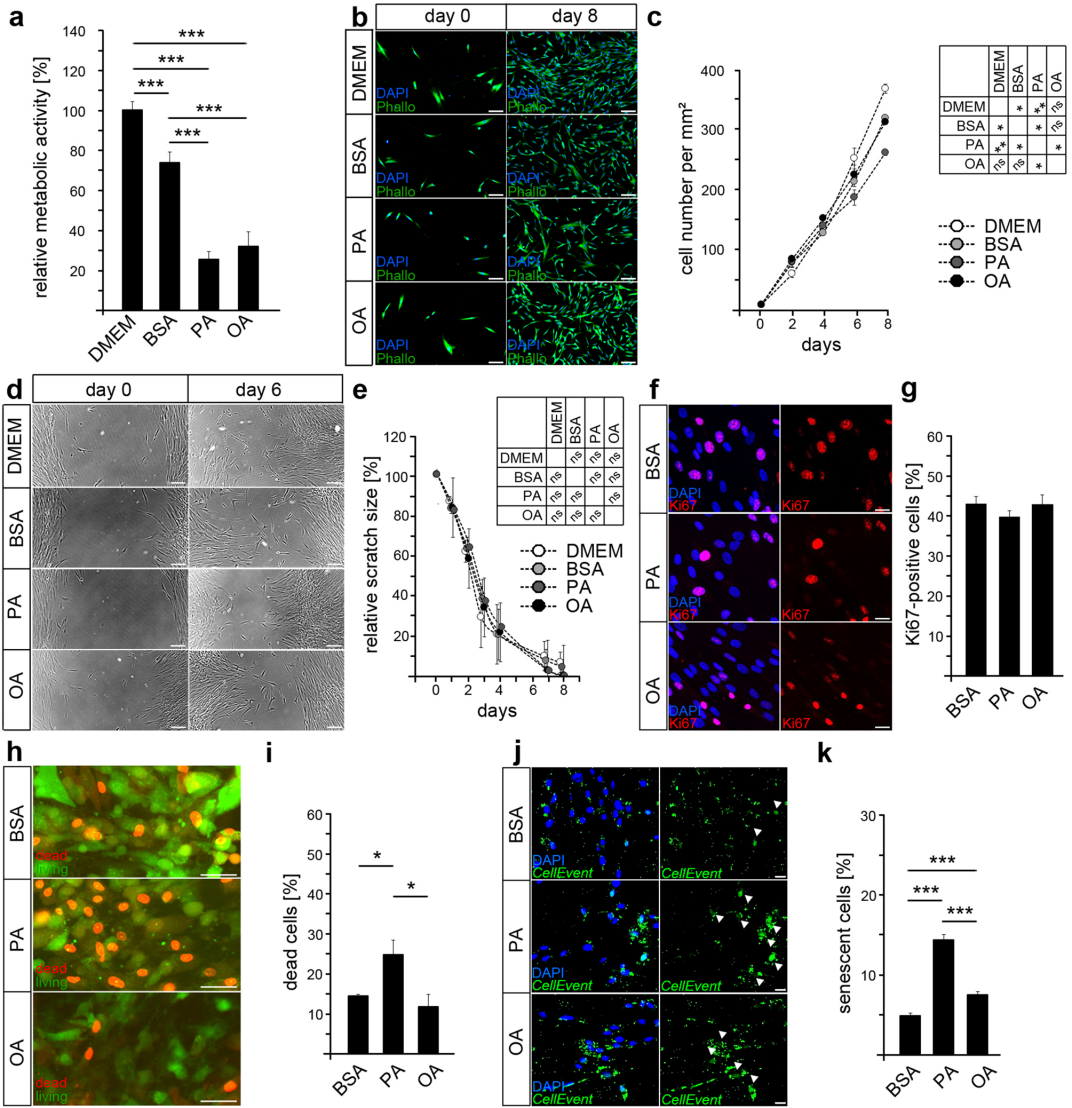
The cell survival of human periodontal ligament fibroblasts is impaired through palmitic acid cultivation. (**a**) Analysis of the metabolic activity of human periodontal ligament fibroblasts (HPdLF) cultured either with DMEM, DMEM containing BSA, palmitic acid (PA) or oleic acid (OA). Both fatty acids were coupled to BSA, which is therefore used as control condition. The metabolic activity is displayed in relation to the DMEM control. (**b**) Representative microphotographs of HPdLF at day 0 and day 8 of culturing with fatty acids with actin cytoskeleton (green) stained with phalloidin-coupled Alexa488 and DAPI-stained cell nuclei (blue). Day 0 is 6 h after cell seeding. Cell number per square millimeter is quantified in (**c**). (**d**) Representative images showing scratch sizes on HPdLF monolayers at day 2 and day 6 after scratch test quantified in (**e**) as relative size to the initial scratch size at day 0 for each fatty acids culture condition. Day 0 is the day the scratch was carried out. (**f**) Representative microphotographs of HPdLF cultured with fatty acids and stained for Ki67 (red). Cell nuclei are visualized by DAPI-staining (blue). The number of Ki67-positive cells in relation to the total cell number is displayed in (**g**). (**h**) Microphotographs of HPdLF cultured with fatty acids and stained for defects in the plasma membrane integrity by EthD-1 (red) referring as dead cells in relation to the total cell number of living cells (green) visualized by their esterase activity. The amount of dead cells was quantified in (**i**) in relation to the total number of living cells. (**j**) Representative microphotographs of HPdLF cultured with fatty acids showing senescent cells (*CellEvent*, green) and an cell nuclei (DAPI, blue). Arrow heads indicate senescent cells, which are analyzed as percentage of DAPI-positive cells in (**k**). For all conditions, cells from biological triplicates were analyzed. *P < 0.05; **P < 0.01; ***P < 0.001; One-Way ANOVA and post-hoc test (Tukey). Photographs were analyzed with *Fiji* software (https://imagej.net/Fiji). Scale bars: 50 μm in (b, d), 20 µm in (h), 10 μm in (f, j). BSA, bovine serum albumin; HPdLF, human periodontal ligament fibroblast; OA, oleic acid; PA, palmitic acid.

To investigate the influence of palmitic and oleic acid on cells of the periodontium, we treated human periodontal ligament fibroblasts (HPdLF) with 200 µM PA or 200 µM OA over six days and analyzed their metabolic activity via colorimetric MTT assay (Fig. 1a). These concentrations were used in previous studies^30^ and are typically found in this ratio to serum albumin in obesity and hypertriglyceridemia^23–28^. Cells with an active metabolism convert MTT to violet formazan, which is commonly used to analyze relative cell numbers. We detected significantly reduced metabolic activity levels in both, PA- and OA-treated HPdL-fibroblasts compared to BSA-treated controls. BSA was used as a cell membrane-passing carrier that binds free fatty acids such as PA and OA^17,21,22^. Since several studies reported BSA-based cellular effects on specific molecular parameters, HPdLF grown in normal DMEM culture medium were additionally added as untreated controls. In BSA-treated HPdLF, metabolic activity was reduced by 25.99% ± 5.26 as compared to untreated DMEM controls.

To verify changes in HPdLF-specific cellular growth, we analyzed cell numbers over eight days in fatty acid containing media starting 24 h after seeding on well-plates (Fig. 1b,c). Compared to BSA controls, increase in cell number of PA-cultured HPdLF was significantly less, whereas OA-treated cells showed no altered growth rate. In addition, we detected a slightly reduced increase in cell number of BSA-treated cells compared to DMEM controls.

The denser HPdLF grow, the more they show parallel arrangements with intensive cell–cell contacts and they start building calcified nodules^35,36^. For further investigation of growth characteristics of dense HPdLF, we performed a scratch assay on complete monolayers of HPdLF grown for one week in DMEM prior six-day fatty acid cultivation (Fig. 1d,e). However, neither PA- nor OA-treated cells showed differences in comparison to the controls when analyzing the time that was required to close the scratch. In summary, these data show that although PA negatively affects the increase in cell numbers during treatment, it does not affect the wound healing capacity of confluent cells.

Differences in cell numbers can correlate with reduced cell proliferation. We asked, whether PA affect the proliferation of HPdLF using Ki67, a well characterized marker for proliferating cells. However, immunohistochemical staining of HPdLF revealed no significant differences in the number of Ki67-positive cells after six days of both fatty acid treatments compared to BSA controls (Fig. 1f,g).

In order to test whether the reduced cell growth of PA-treated cells is due to an altered cell viability, we carried out a cytotoxicity assay discriminating between living and dead cells (Fig. 1h,i). Compared to BSA controls, we found an increased number of PA-treated cells with intracellularly localized red-colored EthD-1, indicating a loss of plasma membrane integrity. In contrast to that, cultivation with OA had no effect on cell survival pointing to relevant differences in the impact of both fatty acids on the growth and viability of HPdLF. Thus, our findings further support a cell survival-regulating effect of PA as cause for decreased cell growth.

Since the metabolic activity was also reduced in OA-treated cells but cellular proliferation and cell numbers were not significantly different to BSA controls, we further investigated potential reasons. Thus, we analyzed whether cellular senescence could be a cause for the diminished metabolic activity. In fact, we noticed a slight increase in OA-treated cells as compared to BSA controls, however, treatment with PA resulted in a more pronounced increase in senescent cell numbers (Fig. 1j,k).

Therefore, our data emphasize that palmitic acid in particular negatively affects cell activity and survival by inducing cellular senescence and cell death. In addition, oleic acid also appears to have a slight impact on cell activity, but not on cell survival.

### Oleic acid, but not palmitic acid, promotes the osteogenic differentiation of PdLF

**Figure 2:**
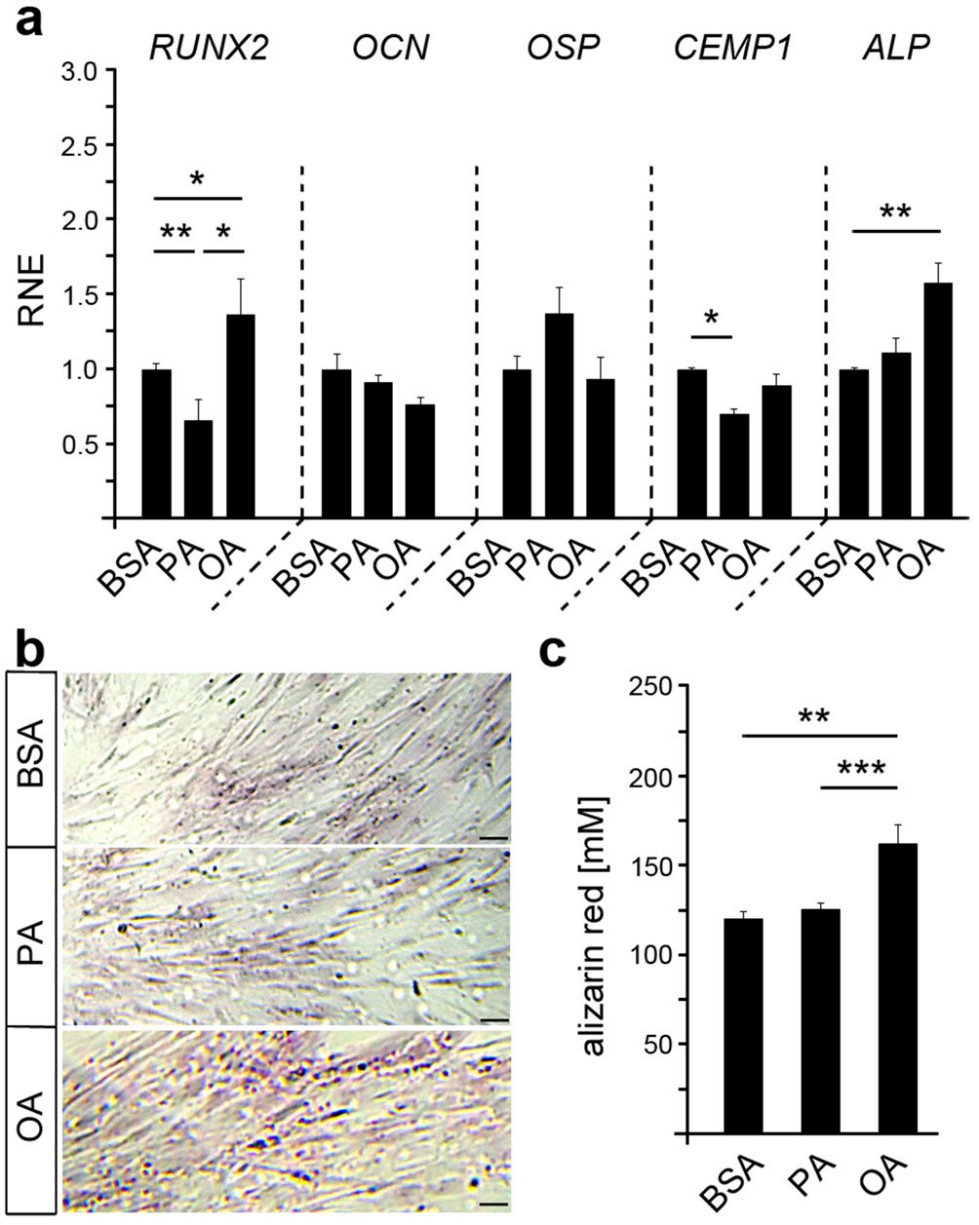
Oleic acid promotes the osteogenic differentiation of HPdLF. (**a**) Quantitative expression analysis of the osteogenic marker genes *RUNX2*, *osteocalcin* (*OCN*), *osteopontin* (*OSP*), *cementum protein 1* (*CEMP1*) and *alkaline phosphatase* (*ALP*) in fatty acid-cultured HPdLF compared to BSA controls. (**b**) Representative microphotographs of alizarin red staining of calcium deposits in HPdLF cultured in fatty acids and displayed in (**c**). *P < 0.05; **P < 0.01; ***P < 0.001; One-Way ANOVA and post-hoc test (Tukey). Scale bars: 50 μm in (b). BSA, bovine serum albumin; OA, oleic acid; PA, palmitic acid; RNE, relative normalized expression.

To investigate whether an enhanced fatty acid exposure affect osteogenic differentiation of HPdLF, quantitative expression analysis of relevant osteogenic marker genes (*RUNX2*, *OCN*, *OSP*, *CEMP1*, *ALP*) was performed on cells that had been treated for six days. We detected an increased expression of the early osteogenic markers *RUNX2* and *ALP* in OA-treated cells, whereas PA-treated cells showed rather reduced levels of *RUNX2* and *CEMP1* (Fig. 2a). Therefore, we assume that OA-treated HPdLF were in an early stage of osteoblastic differentiation since the expression of the late marker genes for osteogenic differentiation *OCN* and *OSP* were not increased.

To further test for osteogenic differentiation of HPdLF, we performed alizarin red staining of calcium deposits in cell cultures that had been treated with fatty acid for six-days (Fig. 2b,c). Even with a relatively weak staining intensity, possibly due to the short cultivation time of the cells, we could detect an increase in the OA-treated HPdLF of 34.79 ± 8.51% as compared to BSA controls, while PA did not induce any changes. This emphasizes an increase in osteogenic differentiation of OA-treated HPdLF. Since differentiated cells often show reduced metabolism of MTT^37^, this could be another reason for diminished metabolic activity levels in OA-treated cells (Fig. 1a). Overall, our data indicate that OA seems to promote the differentiation of periodontal ligament fibroblasts into osteoblasts, whereas PA appears to have no impact on osteogenic cell fate.

### Palmitic acid affects the transcription of MMPs relevant for tissue remodeling processes induced by HPdLF in response to mechanical stimulation

**Figure 3:**
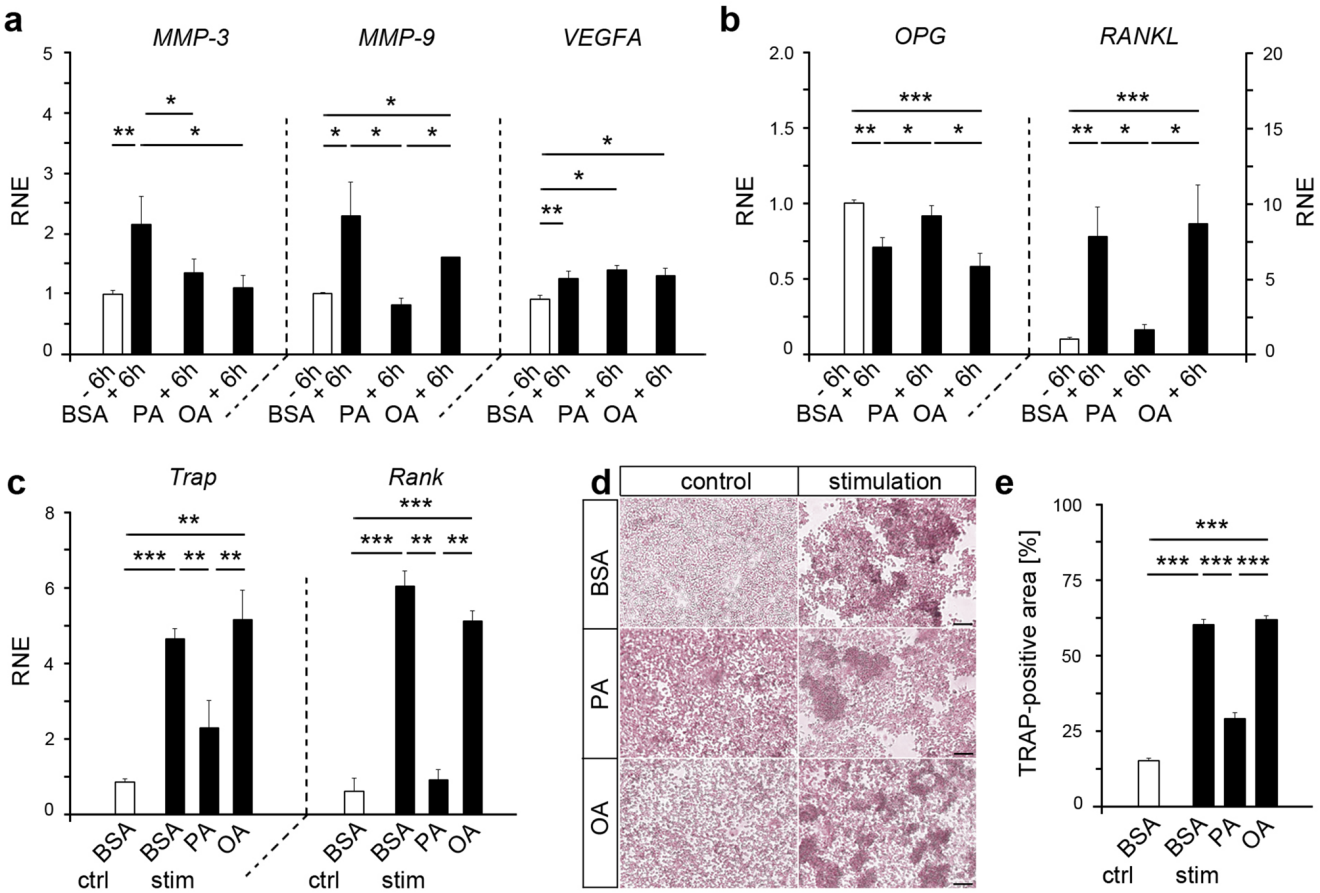
Fatty acid cultivation of mechanically stressed HPdLF affect genes relevant for extracellular matrix degradation as well as influence osteoclast differentiation. (**a**) Quantitative expression analysis of extracellular matrix (ECM)-remodeling genes *MMP-3* and *MMP-9* as well as *VEGFA* coding for an vascularization factor in HPdLF treated with palmitic acid (PA) or oleic acid (OA) and stimulated with compressive force over six hours compared to unstimulated BSA controls. (**b**) Quantitative expression analysis of osteoclastogenesis-relevant genes *OPG* and *RANKL* in HPdLF treated with fatty acids and stimulated with compressive force over six hours compared to unstimulated BSA controls. (**c**) Quantitative expression analysis of osteoclast-related genes *Trap* and *Rank* in mouse osteoclasts stimulated with the media supernatant of mechanically compressed HPdLF cultured in fatty acids compared to BSA (stim BSA, stim PA, stim OA) and only BSA-containing media as control (ctrl BSA). The results are normalized to ctrl BSA. (**d**) Representative microphotographs of TRAP-staining of osteoclasts stimulated with the medium supernatant of fatty acid treated and mechanically compressed HPdLF quantified as TRAP-positive area of the total area in (**e**). *P < 0.05; **P < 0.01; ***P < 0.001; One-Way ANOVA and post-hoc test (Tukey). Photographs were analyzed with *Fiji* software (https://imagej.net/Fiji). Scale bars: 50 μm in (c) Ctrl, control; BSA, bovine serum albumin; HPdLF, human periodontal ligament fibroblast; OA, oleic acid; PA, palmitic acid; RNE, relative normalized expression; stim, stimulation.

For mechanical force-induced tooth movement, remodeling of the extracellular matrix as well as reorganization of blood supply is necessary^38,39^. We therefore asked whether overexposure to palmitic and oleic acid affects the expression of relevant regulators of these processes in mechanically stimulated HPdLF. For this purpose, we analyzed quantitative expression of genes coding for the *matrix metalloproteinase 3* (*MMP-3*) and *MMP-9* as well as for the *vascular endothelial growth factor A* (*VEGFA*) (Fig. 3a). All genes were up-regulated in mechanically stimulated BSA-treated controls. Whereas exposure to OA only affected *MMP-3* expression, PA-treated HPdLF revealed no increase in *MMP-3* and *MMP-9* upon mechanical compression (Fig. 3a). Mechanically induced changes in the transcription *VEGFA* were not changed upon fatty acid treatment. Together, our data suggests fatty acid-dependent changes in the up-regulation of important MMPs, which could affect the degradation on the extracellular matrix due to mechanical stimulation.

### Enhanced fatty acid exposure impacts the modulation of osteoclastogenesis by HPdLF

During orthodontic tooth movement (OTM), mechanically stimulated HPdLF show reduced OPG and increased RANKL expression and secretion on the compressed side promoting osteoclastogenesis^33^. Whether fatty acids impact the regulation of these key players during OTM was assessed by quantitative expression analysis of PA- and OA-treated HPdLF after 6 h of mechanical compression (Fig. 3b). In basic unstimulated conditions, we did not detect significant differences in *OPG* and *RANKL* expression in PA- and OA-treated cells compared to BSA controls (data not shown). However, a significant *OPG* reduction and an increase in *RANKL* found in mechanically-stressed BSA controls as well as in OA-treated HPdLF were not apparent in PA-treated HPdLF (Fig. 3b). This emphasizes that enhanced exposure to PA could hamper typical transcriptional responses of HPdLF regulating osteoclast differentiation or activation in response to compressive stimuli.

To test this, we stimulated immature osteoclasts (OC) isolated from mice hind and front limbs with the supernatant of fatty acid-cultivated and mechanically stimulated HPdLF over six days, respectively. To rule out HPdLF-independent effects on cell differentiation caused only by the cultivation with fatty acids, additional control cultures were performed using fatty acid-containing media only. First, we quantitatively analyzed the expression of two key players of differentiating osteoclasts coding for the *Tartrate-resistant acid phosphatase (Trap*) and the *receptor activator of nuclear factor-kappaB* (*Rank*). Osteoclasts stimulated with the supernatant of mechanically stressed HPdLF cultured in OA showed comparable levels of *Trap* and *Rank* expression as in the control stimulation with the supernatant from mechanically stressed BSA-cultured HPdLF (Fig. 3c). These expression levels were significantly increased compared to the BSA control (Fig. 3c). In contrast to that, we detected significantly reduced *Trap* and *Rank* expression in OC that were stimulated with the supernatant of mechanically stressed PA-HPdLF cultures compared to the stimulated BSA condition (Fig. 3c). However, *Trap* expression was still significantly increased as compared to the BSA control condition indicating an up-regulation through mechanical compression. In addition, no differences were found in control cultivation with only PA or OA (data not shown).

To further analyze the differentiation of immature osteoclasts, we performed a tartrate-resistant acid phosphate activity (TRAP) assay of cultured OC. Our results show a significantly reduced area of TRAP-positive cells upon treatment with media from mechanically stressed HPdLF cultured in PA (Fig. 3d,e) whereas OA-treated cultures presented a comparable amount of TRAP staining as the BSA control. Effects of media containing only fatty acids was not detected (data not shown). Our results emphasize that mechanically stressed PA-HPdLF cultures secrete less factors driving osteoclast differentiation as compared to OA-cultures.

All together our data indicate that the fatty acids palmitic and oleic acid have a strong impact on cell metabolism, survival and function of human periodontal ligament fibroblasts under control condition and mechanical stimulation. Whereby oleic acid seems to support bone assembly by stimulating the osteogenesis of HPdLF, palmitic acid rather appears to induce cell death of HPdLF as well as inhibit typical processes driving bone degradation.

## Discussion

Obesity is a high-prevalence global health problem that can lead to serious medical problems such as diabetes, hyperlipidemia, cardiovascular disease, chronic inflammation and changes in bone metabolism^40,41^. A direct link between obesity and alveolar bone loss was already reported in animal studies as well as in the analysis of patient data, especially in the context of inflammatory periodontal disease^12–14,42^. Thereby, a high-fat diet inducing obesity was shown to trigger alveolar bone loss by disturbing physiological bone remodeling^29,30,42,43^. Impairments in bone remodeling can be associated with diseases like osteoporosis and osteopetrosis in general^44,45^ as well as with specific risks during orthodontic procedures like tooth root resorption or tooth loss^6^.

In this study, we investigated the impact of differential fatty acid overexposure on the function of periodontal ligament fibroblasts, a major cell type regulating bone remodeling and inflammation in response to mechanical stimulation^2–4^. Fatty acids are essential for normal cell function as they are substantial components of lipid vesicles and phospholipids in cell membranes^46^. In the case of obesity, an increased serum level of circulating fatty acids is in particular a result of the release from the enlarged adipose tissue mass and can influence cell metabolism^15,16^. In line with this, negative effects of the saturated lipid acid palmitate on cell metabolism and survival of osteoblasts and other cell types have already been reported in several studies^17–20^. On the other hand, a positive impact could be detected for oleate in different cell types^17,21,22^. Our results show a reduced amount of metabolic activity in HPdLF under both hyperlipidemic fatty acid cultivation conditions, which may be partly due to the increase in the number of senescent cells. Additionally, palmitate-cultured HPdLF also showed reduced cell growth and higher cell death rates as a possible cause for a detected reduced metabolic activity. Overexposure with palmitate was already reported to induce cellular senescence^47^, whereas oleate is generally seen to promote longevity of cells^48,49^. However, in neural stem cells enhanced exposure to oleic acid reduced cell proliferation^50^ indicating that increasing oleate concentrations might not be beneficial in all cells and tissues. Apart from this, several studies reported that already a slight increase in intracellular palmitate concentration has been shown to be toxic to mitochondria and the endoplasmic reticulum with ultimately resulting in apoptosis^51–54^. In addition, it has been reported that fatty acid metabolites of cycloxygenase, lipoxygenase and cytochrome P450 are involved in programmed cell death^55^.

Interestingly, differentiation processes can also have crucial effects on the metabolic activity of cells^37^, which possibly explains the changes in oleate-cultured HPdLF since we found an increase in osteogenic differentiation of HPdLF in response to oleate treatment. It already had been reported that HPdLF show a distinct osteogenic differentiation potential^31,32,56^, which can be stimulated by several external cues^35,57–59^. In addition, fatty acids can affect the differentiation of osteoblasts^60^. However, how they influence HPdLF was unknown so far. We detected increased expression of the osteogenic marker genes *RUNX2* and *ALP* in oleate-cultured HPdLF, whereas palmitate treatment reduced the expression of *RUNX2* and *CEMP1*. Moreover, alizarin red staining of calcium deposits revealed enhanced levels in oleate-cultured HPdLF. Since we analyzed cells after six days of culturing, osteogenic differentiation in oleate-treated HPdLF was potentially in an early phase, which is supported by the increased expression of only early osteogenic markers (*RUNX2*, *ALP*), but not late markers (*OCN*, *OSP*). However, some oleate-treated cells seem to have already formed calcium depots, which was evident from the alizarin red staining.

These results are consistent with recent studies, which show that increased exposure to oleic acid may be a potential promoting factor for bone formation^61^, whereas higher palmitic acid levels reduces circulating bone marker in obese animals and impairs osteoblastic activity^29^. One possible pathway in fatty-acid dependent modulation of osteogenesis could be the regulation of SIRT1, a member of the sirtuin protein family that has been reported to stimulate RUNX2 expression^62^. In contrast to palmitate, which was shown to down-regulate SIRT1 expression^63^, oleic acid could activate SIRT1^64^ and thereby promote the osteogenic differentiation in HPdLF. In addition, SIRT1 was also shown as a negative regulator of apoptosis^65^, which further supports our data, since the treatment with palmitate indeed increased cell death in HPdLF. However, palmitic acid was also reported to induce osteoblast differentiation in smooth muscle cells^66^, which emphasizes a differential impact of fatty acids based on cellular background as well as treatment conditions.

Negative effects of palmitate on alveolar bone remodeling were reported in recent animal and in vitro studies^29,30^. Thereby, mice fed with a diet containing high amounts of palmitic acid showed increased osteoclast numbers and activity, while osteoblast mineralization was reduced^29,30^. This could significantly affect bone remodeling due to mechanical stimulation during mastication as well as during orthodontic treatment.

Normally, mechanically compressed HPdLF show a reduced expression of OPG and increased expression and secretion of RANKL^33^, which could be translated into increased osteoclastogenesis on the compressive side of mechanically stimulated teeth^2–4^. We detected similar changes in the *OPG/RANKL* expression in mechanically compressed HPdLF cultured with oleic acid, whereas palmitic acid treatment seemed to block the typical transcriptional changes. In liver cells, it was already reported that palmitate affect the expression of several genes^67^. Profiling mRNA expression revealed changes in the transcription of genes involved in cell growth and proliferation, cell signaling, lipid and cholesterol transport and catabolism as well as in oxidative stress response. But also outside the liver, lipid metabolism was linked to gene expression regulation^68,69^. We did not analyze protein secretion but tested the impact of proteins secreted by HPdLF on differentiation of immature osteoclasts isolated from mice hind and front limbs. While oleic acid appears to promote the typical secretion of osteoclastogenesis-modulating factors by mechanically stressed HPdLF, palmitic acid does not appear to do so. Our data suggest that palmitate treatment rather induce cellular senescence and cell death as cause for the diminished secretion of osteoclastogenesis-modulating factors. In particular, cells that are compromised in their survival due to external cues may not be able to properly perform their regulatory functions due to changes in their metabolism as well as their morphology and cellular contacts^70–72^.

However, these results seems to be in contradiction with studies showing that palmitic acid enhances osteoclastic differentiation and activity^30^. Although we used a comparable amount of BSA-coupled fatty acids in our in vitro studies as could be detected in obese patients^23–28^, side effects due to a different absorption and availability of the fatty acids in the cells cannot be excluded. Further studies on the bone resorption activity of TRAP-positive cells as well as in vivo studies can be carried out to obtain more information about the complex regulation of osteoclastogenesis by PdLF.

Interestingly, we did not detect direct changes in osteoclast differentiation when we treated these cells with fatty acid-containing media, used as controls in our experiments. This could be due to the fact that the control media as well as the ones collected from mechanically stressed HPdLF were initially frozen in aliquots and then thawed for medium change.

Besides their role in modulating alveolar bone remodeling, periodontal fibroblasts are involved in the degradation and assembly of the extracellular matrix (ECM) as well as contribute to the vascularization of the surrounding tissue. Mechanical stress affects ECM degrading matrix metalloproteinases as well as factors regulating the vascularization^2–4^. We detected changes in the expression of *MMP-3/9* in response to both fatty acids, which are in line with several studies that had already reported the impact of palmitate and oleate on MMP expression, activation and secretion^73–76^.

Altogether, our study provides new information on how obesity-associated changes like an increased level of free circulating fatty acids could impact the growth, survival, differentiation and function of human periodontal ligament fibroblasts, a major cell type in alveolar bone remodeling due to their response to mechanical forces. In addition to the previously detected direct effects of fatty acids on osteoclast and osteoblast differentiation, our results further revealed indirect effects mediated by mechanically stressed HPdLF generating a link between bone remodeling by periodontal ligament cells, obesity and orthodontic tooth movement. Up to now, there is only a small number of studies addressing possible effects of obese conditions in orthodontic treatment^77–79^. However, along with the increasing number of obese patients, the interest in orthodontic treatment is also growing. Problems associated with obesity could therefore increase the risks occurring during orthodontic procedures, since duration and outcome of the treatment dependent to a large extent on individual conditions such as the bone remodeling capacity and the quality of newly formed bone as well as the inflammatory status. Further research is necessary to investigate the complex interplay between periodontal ligament cells, osteoclasts as well as osteoblasts in alveolar bone remodeling of obese patients to address potential orthodontic problems like tooth root resorption and tooth loss caused by therapeutic interventions.

## Material and methods

### Animals

All animal procedures and experimental protocols were approved by the local government and licensing institution (Thueringer Landesamt für Verbraucherschutz, TLV Abteilung 5, Bad Langensalza, Germany) and performed in strict compliance with the EU directives 86/609/EWG and 2007/526/EG guidelines for animal experiments. Animals were housed in plastic cages under 12 h light/dark conditions with ad libitum access to food and water. NC3Rs ARRIVE guidelines were followed^80^.

### Osteoclast preparation

For analysis of osteoclast differentiation, two four-weeks old C57Bl6 mice were deeply anesthetized by CO_2_, sacrificed by cervical dislocation and bone marrow was isolated by centrifugation of prepared front and hind limbs according to Amend et al.^81^ and incubated in a density of max. 1 × 10^8^ cells on a 10 cm dish overnight in proliferation medium (MEMα (Sigma) containing 10% fetal bovine serum albumin (FBS, Gibco) and 1% penicillin/streptomycin) according to Cappellen et al.^82^. Following day, non-adherent osteoclast precursors were seeded in a density of 1.2 × 10^5^ cells per well and grown in at 37 °C, 5% CO_2_ and 95% humidity prior further treatment.

### Cell culture

Human periodontal ligament fibroblasts (HPdLF, Lonza) were cultured in Dulbecco’s modified Eagle’s medium (DMEM; Invitrogen) containing 4.5 g/L glucose, 1% L-glutamine, 10% FBS (Gibco), 1% penicillin/streptomycin and 1% L-ascorbic acid at 37 °C, 5% CO_2_ and 95% humidity. All cells were passaged regularly using either 0.05% Trypsin/EDTA (Invitrogen) or Accutase (Merck Millipore) and used for experiments at passages four to eight.

### Fatty acid stimulation in HPdLF

Palmitic and oleic acid were solved in water containing 50 mM NaOH at 70 °C and then bound to preheated 37 °C bovine serum albumin (BSA, H2B) before adding to the culture medium (DMEM, 10% FBS 1% penicillin/streptomycin, 1% L-ascorbic acid). The final concentration of BSA was 0.66% in culture medium and the fatty acid concentration was adjusted to 200 µM. Stimulation of HPdLF with either fatty acids (FA) or BSA as control was performed for six days.

### Mechanical strain devices

Prior mechanical stimulation, 2.5 × 10^4^ HPdLF were seeded randomly on 6-well cell-culture plates and cultured for six days in fatty acid-containing media resulting in a 65–75% confluent state. A compressive force of 2 g/cm^2^ was applied via the application of sterilized glass plates for 6 h comparable to the 24 h-protocol of Kirschneck et al.^83^. For RNA analysis, cells were isolated with TRIzol Reagent (Thermo Fisher Scientific) 6 h after compressive strain application.

### RNA extraction and quantitative reverse transcription (RT) PCR

For gene expression analysis of HPdLF, RNA was isolated and analyzed according to Symmank et al.^84^. Briefly, standard phenol–chloroform extraction was performed and RNA was purified with *RNA Clean & Concentrator-5 kit* (Zymo Research). Synthesis of cDNA was performed with *SuperScript III Reverse Transcriptase* (Invitrogen) according to manufacturer`s protocols. Quantitative RT-PCR was performed with *Luminaris Color HiGreen qPCR Master Mix* (Thermo Fisher Scientific) and analyzed with qTOWER3 (Analytik Jena).

Primers sequence information are displayed in Table 1 for primers directed against human gene targets and in Table 2 for primers against mouse targets.

**Table 1:** qPCR primer sequences indicated in 5´–3´ direction for human gene targets with gene symbols and alternative names used in the manuscript (fw as forward, rev as reverse).

Gene	Gene symbol	NCBI accession number	Primer sequence
Alkaline phosphatase	*ALP*	NM_000478.6	fw ACTGCAGACATTCTCAAA rev GAGTGAGTGAGTGAGCA
Cementum protein 1	*CEMP1*	NM_001048212.3	fw GCCAAGGGGACACAGAAGAT rev GGTGCTGGAGGGCAGTATG
Matrix metallopeptidase 3	*MMP-3*	NM_002422.5	fw ACCCTTTTGATGGACCTGGA rev GGCTGAGTGAAAGAGACCCA
Matrix metallopeptidase 9	*MMP-9*	NM_004994.3	fw ATTTCTGCCAGGACCGCTTC rev TCATAGGTCACGTAGCCCACT
Bone gamma-carboxyglutamate protein	*BGLAP* (alias *OCN*)	NM_199173.6	fw CAGGCGCTACCTGTATCA rew CTGGAGTTTATTTGGGAG
Secreted phosphoprotein 1	*SSP1* (alias *OSP*)	NM_001040058.2	fw TGATTTTCCCACGGACCTGC rev TCGCTTTCCATGTGTGAGGT
TNF receptor superfamily member 11b	*TNFRSF11B* (alias *OPG*)	NM_002546.4	fw GAAGGGCGCTACCTTGA rev GCAAACTGTATTTCGCTC
TNF Superfamily Member 11	*TNFSF11* (alias *RANKL*)	NM_003701.4	fw ATCACAGCACATCAGAGCAGA rev TCACTTTATGGGAACCAGATGGG
RUNX family transcription factor 2	*RUNX2*	NM_001015051.3	fw CCCACGAATGCACTATCC rev GGACATACCGAGGGACA
Ribosomal protein L22	*RPL22*	NM_000983.4	fw TGATTGCACCCACCCTGTAG rev GGTTCCCAGCTTTTCCGTTC
TATA-box binding protein	*TBP*	NM_003194.5	fw CGGCTGTTTAACTTCGCTTCC rev TGGGTTATCTTCACACGCCAAG
Vascular Endothelial Growth Factor A	*VEGFA*	NM_001171623.1	fw TCCAGGAGTACCCTGATGAGAT rev TGGTGAGGTTTGATCCGCATA

**Table 2:** qPCR primer sequences indicated in 5´-3´ direction for mouse gene targets with gene symbols and alternative names used in the manuscript (fw as forward, rev as reverse).

Gene	Gene symbol	NCBI accession number	Primer sequence
Glyceraldehyde 3-phosphate dehydrogenase	*Gapdh*	NM_001289726.1	fw TGTGAACGGATTTGGCCGTA rev ACTGTGCCGTTGAATTTGCC
Tumor necrosis factor receptor superfamily, member 11a, NFKB activator	*Tnfrsf11a* (alias *Rank*)	NM_009399.3	fw TGCAGCTCAACAAGGATACG rev GTGCAGTTGGTCCAAGGTTT
Ribosomal protein S29	*Rps29*	NM_009093.2	fw GAAGTTCGGCCAGGGTTCC rev GAAGCCTATGTCCTTCGCGT
Acid phosphatase 5, tartrate resistant	*Acp5* (alias *Trap*)	NM_001102405.1	fw CCAATGCCAAAGAGATCGCC rev TCTGTGCAGAGACGTTGCCAAG

Primer quality was assessed by melting curve analysis and agarose gel electrophoresis. Primer efficiency was calculated using tenfold dilution series. RNA levels of compressed HPdLF were normalized to *RPL22* and *TBP* as reference genes according to Kirschneck et al.^83^. *Gapdh* and *Rps29* were used as reference genes for osteoclasts. Data were analyzed with efficiency corrected ΔΔCT method^85^.

### MTT cell vitality test

Cell vitality of HPdLF stimulated with fatty acids was analyzed with MTT colorimetric assay (Sigma Aldrich) according to manufacturer`s protocol and compared with BSA and untreated controls (DMEM).

### Growth and scratch assay

For the analysis of cell growth, HPdLF were seeded with a density of 300 cell per mm^2^ on coverslips 24 h prior fatty acid cultivation. After 2, 4, 6 and 8 days of treatment coverslips were fixed for 10 min in 4% paraformaldehyde. Cells were washed three times in phosphate-buffered saline (PBS), actin cytoskeleton was stained with Phalloidin coupled to Alexa488 (Thermo Fisher Scientific) for 20 min and nuclei were stained 5 min with DAPI (Thermo Fisher Scientific; 1:10,000 in PBS). Coverslips were embedded in Mowiol (Sigma Aldrich).

Further analysis of cell growth was performed by applying a scratch of 0.1 cm width to monolayers of HPdLF grown for seven days in DMEM prior six-day cultivation in fatty acid-containing media. The scratch size reduction was analyzed in bright field images over eight days.

### Cellular senescence assay

HPdLF cultured on coverslips were stained after six days of fatty acid cultivation for senescent cells with the fluorescent *CellEvent Senescence Green Detection Kit* (Thermo Fisher Scientific) according to manufacturer`s protocol. Senescent cells were counted in relation to DAPI cells.

### Life-dead-assay

HPdLF cultured on coverslips were stained after six days of fatty acid cultivation for living and dead cells with the *LIVE/DEAD Viability/Cytotoxicity Kit for mammalian cells* (Thermo Fisher Scientific) according to manufacturer`s protocol.

### ALP activity assay

Alkaline phosphatase activity of HPdLF cultured for six days in fatty acid-containing media was analyzed with *p-Nitrophenyl Phosphate Liquid Substrate System* (Sigma-Adrich Chemie GmbH) according to Lossdorfer et al.^86^.

### Immunohistochemistry

HPdLF cultured on coverslips were fixed in 4% PFA for 10 min after six days of fatty acid cultivation, washed three time in PBS prior primary antibody cultivation for three hours and secondary antibody cultivation for 45 min. Nuclei were stained with DAPI (Thermo Fisher Scientific; 1:10,000 in PBS). Mouse-anti-human-Ki67 (Leica; 1:500) was used to detect Ki67 in HPdLF. Goat-anti-Mouse-Cy3 (Jackson ImmunoResearch; 1:1,000) was used for fluorescent detection and labeling.

### Osteogenesis-assay (alizarin red staining)

For the detection of osteogenic differentiation, HPdLF were treated over six days with fatty acid containing media and fixed in 10% PFA for 15 min prior to alizarin red staining. Calcium deposits were stained with 40 mM Alizarin Red S (Sigma) for 20 min, rinsed in water and dissolved with 10% acetic acid. After heating at 85 °C for 10 min, cells were centrifuged for 15 min at 20,000 g and pH of the supernatant was neutralized with 10% ammonium hydroxide. OD405 was measured with the microplate reader *Infinite M Nano plate reader* (Tecan Life Science) as duplicates.

### TRAP-assay

For the analysis of osteoclast differentiation induced by mechanically stressed HPdLF cultured in PA- or OA-containing media, six days grown immature mouse osteoclasts were culture for further six days in the collected media supernatant of HPdLF as describe above. As control for side effects, thawed FA- and BSA-media was used. Cells were prefixed in 4% PFA for 10 min, then fixed for 1 min in 50:50 acetone/ethanol and stained for TRAP according to Muluke et al.^87^. For the experiment, cells of two animals were pooled, seeded in duplicates for three experiments and each experiment was treated with culture media supernatant of HPdLF collected in three different experiments.

### Microscopy, image analysis and statistics

Images were taken with the inverted confocal laser scanning microscope TCS SP5 (Leica). Photographs were analyzed with *Fiji* software (https://imagej.net/Fiji). Figure illustration was performed with *Adobe Photoshop CS5* (https://adobe.com). One-way ANOVA and post-hoc test (Tukey) were used as statistical tests. Significance levels: *P* value < 0.05 *; *P* value < 0.01 **; *P* value < 0.001 ***. Experiments were repeated three times with two or more replicates per experiment if not stated differently.

## Data Availability

The datasets generated during and/or analyzed during this study are available from the corresponding author on reasonable request.
